# Impacts of Human Disturbance on Large Prey Species: Do Behavioral Reactions Translate to Fitness Consequences?

**DOI:** 10.1371/journal.pone.0073695

**Published:** 2013-09-11

**Authors:** Mathieu Leblond, Christian Dussault, Jean-Pierre Ouellet

**Affiliations:** 1 Département de biologie, chimie et géographie, Université du Québec à Rimouski et Centre d’études nordiques, Rimouski, Canada; 2 Direction de la faune terrestre et de l’avifaune, Ministère du développement durable, de l’environnement, de la faune et des parc du Québec, Québec, Canada; University of Regina, Canada

## Abstract

Anthropogenic disturbances have been demonstrated to affect animal behavior, distribution, and abundance, but assessment of their impacts on fitness-related traits has received little attention. We hypothesized that human activities and infrastructure cause a decrease in the individual performance of preys because of anthropogenically enhanced predation risk. We evaluated the impacts of commercial logging and road networks on the fitness of a large herbivore known to be sensitive to human disturbance: the forest-dwelling woodland caribou (*Rangifer tarandus caribou*). For 8 consecutive years (2004–2011) we monitored 59 individuals using GPS telemetry in the Charlevoix region of Québec, Canada. We also used *Very High Frequency* telemetry locations collected on 28 individuals from 1999–2000. We related habitat selection of adult caribou at various spatio-temporal scales to their probability of dying from predation, and to indices of their reproductive success and energy expenditure. The probability that adult caribou died from predation increased with the proportion of recent disturbances (including cutblocks ≤5 years old) in their annual home range. The respective effects of increasing paved and forestry road densities depended upon the overall road density within the home range of caribou. At a finer scale of 10 to 15 days before their death, caribou that were killed by a predator selected for recent disturbances more than individuals that survived, and avoided old mature conifer stands. The home range area of caribou increased with road density. Finally, the composition of the home range of females had no effect on their reproductive success. We show that human activities and infrastructure may influence the individual performance of large prey species in highly managed regions. We outline the need to consider the full set of impacts that human development may have on threatened animal populations, with particular emphasis on predator-prey relationships and population dynamics.

## Introduction

Human disturbances including industrial development, transportation, or resource extraction may have damaging effects on wildlife [1]. Many studies have investigated changes in behavior, abundance, or distribution of organisms resulting from anthropogenic disturbance (*e.g.*, see reviews by [2]–[3]). However, the direct and indirect impacts of human disturbances on animal fitness have received less attention. Assessing the impacts of human activities on the fitness of free-ranging wildlife requires long-term, spatially explicit observations [4], which are difficult to obtain, especially for long-lived, large mammal species. Yet these species are likely the most vulnerable to extirpation by humans because of their extensive habitat requirements and low reproductive rates [5]. Determining whether behavioral modifications in response to human disturbance translate into impacts on survival or reproductive success is of paramount importance to develop suitable mitigation measures and conservation plans for large mammals [6].

Commercial logging activities and road networks are two major sources of anthropogenic disturbance which may affect individual performance of large mammals that occur in managed forested landscapes. First, vehicle collisions on roads may cause a substantial number of deaths, threatening the persistence of vulnerable populations [7]. Roads may modify foraging routes [8], and create barriers to animal movements [9] and mating opportunities [10]. Commercial logging may cause functional habitat loss and fragmentation [11]–[12], constraining the resource selection of animals towards riskier environments and possibly increasing predation risk [13]. Finally, behavioral changes due to human disturbances (*e.g.*, increased vigilance) may reduce the time and energy allocated to other activities, like foraging or mating, resulting in considerable implications for growth, body weight, and/or reproduction [14]. Thus, animals selecting resources must make trade-offs between habitat patches that are favorable for foraging and breeding activities while avoiding the risks associated with predation and human activities [15].

Recent empirical evidence also suggests that human infrastructure may strongly affect trophic interactions in large mammal communities. James and Stuart-Smith [16] found that wolves (*Canis lupus*) in Alberta (Canada) were able to capture large prey more efficiently in the vicinity of linear corridors. Conversely, Berger [17] observed that female moose (*Alces alces*) in Yellowstone (USA) gave birth closer to roads as brown bear (*Ursus arctos*) density increased in the park, and suggested that moose were using human infrastructure as a shield from their human-averse predators. Therefore, anthropogenic disturbances could have different impacts on animal performance, depending upon the responses of both prey and predator and the scale(s) at which those animals react to human activity [18]–[19].

In this study, we investigated the consequences of major landscape disturbances on various fitness-related traits in a large herbivore that was previously shown to display strong behavioral reactions to human infrastructure: the forest-dwelling (boreal) caribou (*Rangifer tarandus caribou*). Caribou are declining across most of their range [20] and forest-dwelling caribou, a woodland caribou ecotype that occurs in the North American boreal forest, are listed as threatened under Canada’s Species At Risk Act. The conversion of old-growth conifer forests, forest-dwelling caribou’s prime habitat, into early seral stages of mixed forests following commercial logging [21], as well as the presence of anthropogenic features like road networks [9], are recognized as two major human-induced habitat modifications impeding caribou population recovery. Despite the scarcity of empirical evidence (but see [13], [22]), it is generally thought that anthropogenic sources of disturbance (*e.g.*, cutblocks, linear features) act synergistically to affect caribou fitness.

We investigated the impacts of human disturbances on individual performance of adult caribou, by relating resource selection with proxies of fitness over several spatio-temporal scales. We hypothesized that anthropogenic sources of disturbance cause a decrease in fitness for large prey species, because of the anthropogenically enhanced predation risk (*sensu* [23]). We predicted that predation mortality would increase in cutblocks and regenerating stands following logging where wolves actively search for moose, but also prey on caribou [24]. We also predicted that predation mortality would increase in areas of high road density, because wolves may actively use them as travel routes to patrol their territory and improve their probability of finding and capturing moose, which may also increase their probability of capturing caribou [16].

In addition to survival, fitness is also linked to reproductive success [25] and the amount of energy allocated to activities like foraging [26] and reproduction [27]. To consider these aspects of individual performance in adult caribou, we investigated the impacts of human disturbances on the annual calving rate of females and the probability that a calf died by predation during its first year of life. We also assessed indirect indices of energy expenditure related to patterns of space use by caribou, namely annual and seasonal home range sizes. We predicted that the calving rate of adult females and calf survival would decrease in disturbed areas (*i.e.*, areas of high cutblock and road densities), because of both direct (*i.e.*, calves have a higher probability of being killed by a predator) and indirect costs (*i.e.*, females may allocate less energy to reproduction) of the enhanced predation risk next to human infrastructure [28]. We also predicted that adult caribou would have larger home ranges [29] when using disturbed areas in order to access suitable resources while avoiding human disturbances and predators.

## Materials and Methods

### Ethics Statement

Caribou capture and handling procedures were approved by Animal Welfare Committees of the Ministère des Ressources naturelles et de la Faune du Québec (MRNF) and the Université du Québec à Rimouski (permits CPA#04-00-02 to CPA#10-00-02, renewed each year). Once an animal was caught in the net, the capture team quickly placed a blindfold on its eyes. Animals were not anesthetized, and were immediately released after being fitted with the collar (which took approximately 15 minutes). All manipulations were performed by experienced wildlife technicians. No permit was required in regards to land access, because our field teams were employees of the ministry of natural resources and wildlife.

### Study Area

Our study area (approximately 7 250 km^2^) was located north of Québec City in the Laurentides Wildlife Reserve, Québec, Canada. The study area also included the Grands-Jardins National Park of Québec, initially designed to preserve the critical wintering range of the Charlevoix caribou population, as well as parts of the Jacques-Cartier and Hautes-Gorges-de-la-Rivière-Malbaie National Parks of Québec. Caribou hunting is prohibited in the study area, while commercial logging is allowed in the wildlife reserve but is prohibited in the parks. The region was subject to a high degree of anthropogenic habitat alteration, with approximately 37% of the study area covered by disturbed habitats including roads and numerous cutblocks of different ages (see [30] for additional details and map).

### Caribou Capture, Telemetry, and Data Collection

Between April 2004 and March 2011, we captured 59 caribou (42 female and 17 male) by net-gunning from a helicopter, and fitted them with GPS telemetry collars (models TGW-3600 or TGW-4600, Telonics Inc., Mesa, AZ, USA) programmed to collect locations every 2.5, 3 or 7 hours depending upon the collar model and year. We captured both juveniles (1.5–2.5 years old) and adults (>2.5 years old), and estimated their age using visual examination of tooth wear [31]. We recaptured caribou periodically (at 1 or 2 year intervals) to download location data and replace battery packs. Individuals were monitored for 2 to 81 months. We collected >460 000 caribou locations based on 197 caribou-years, for an average of 2 288 locations per individual, per year. Collars were programmed to drop at the end of the study (winter 2012) with a timer release mechanism. GPS collars were equipped with mortality sensors that transmitted a mortality signal after 4 hours of immobility. We conducted telemetry flights regularly to identify dead individuals and defective collars. The frequency of telemetry flights was ranging from successive flights close in time (*e.g.*, every day during periods of highest calf vulnerability from 2004–2006, see [32]) to a maximum of 3 months apart.

To increase our sample size, we used caribou locations from *Very High Frequency* (VHF) telemetry collars collected on 28 individuals (27 female and 1 male) in the same population from late 1998 to early 2001 [33]. During this period, caribou were located by plane or helicopter every 3 to 5 days during calving (21 May–20 June), rut (15 September–31 October) and late winter (1 February–15 March), and every 3 to 4 weeks during the remainder of the year. This additional database added two complete years of monitoring (1999–2000; 43 caribou-years) to our annual survival analysis. These caribou locations were only used for broader scale analysis because location frequency and accuracy provided by the VHF monitoring was not adequate for other purposes.

When we detected a mortality signal, we visited the site as soon as possible (usually the same day but up to 5 days later) to determine the cause of mortality. We considered the presence of disarticulated, dispersed, or crushed bones, blood, and other signs of predator presence including feces, tracks, hair, tooth marks, and scratching near the carcass as evidence of predation [33]. Because we were interested in mortality by predation, we performed our initial survival analyses only for individuals that died from predation, and “censored” (*sensu* [34]) caribou that died from natural or accidental causes not related to predation. Preliminary analyses performed using individuals which died from an undetermined cause and individuals killed by predators yielded similar results. We also performed preliminary analyses showing that the age and sex of dead individuals, the timing of death, and the habitat characteristics found in a 1-km spatial extent around mortality sites did not differ between confirmed predation events and mortalities of undetermined causes (Table S1). Therefore, and because it is very likely that most undetermined causes of caribou mortality were due to predation (*i.e.*, predation is the main cause of adult mortality in large herbivores [35]), we combined mortalities due to predation and undetermined causes in statistical analyses.

We obtained reproduction data from a companion study that evaluated the reproductive success of all female caribou monitored in the same population from 2004–2007. During this 4-year period, Pinard et al. [32] visually located radio-collared females by helicopter during the calving period to determine if they had given birth. They captured newborn calves, fitted them with an expandable VHF collar (model M2510B, Advanced Telemetry Systems, Isanti, MN, USA), and conducted telemetry flights every day during the first 5 weeks following birth to monitor calf survival. After this period of high calf vulnerability, telemetry flights were spaced 2 months apart, and all calves that survived through the vulnerability period reached at least 1 year-old. Therefore, from 2004 to 2007, we knew whether a female 1) did not calve, 2) had a calf that died soon after birth, or 3) had a calf that survived up to 1 year-old.

### Spatial Data

We determined land cover types using digital forest maps provided by the MRNF, updated annually to include new cutblocks and roads. We created 9 habitat classes based on dominant cover type, tree height and age class. Habitat classes included old mature conifer-dominated forests (conifer and mixed stands >90 years old; availability = 11.5% of the study area), young mature conifer-dominated forests (conifer and mixed stands 50–90 years old; 31.0%), mature deciduous forests (>50 years old; 2.8%), recent cutblocks or natural disturbances (≤5 years old; 10.7%), old cutblocks or natural disturbances (6–20 years old; 10.5%), regenerating stands (generally 20 to 30 years after disturbance; 25.6%), open lichen woodlands (1.0%), wetlands (2.3%), and other (*e.g.*, lakes and powerline right-of-ways; 4.6%). We combined natural disturbances (*e.g.*, forest fires) with anthropogenic disturbances based on their age to reduce the number of habitat classes. Cutblocks were 2.4–3.2 times more abundant than natural disturbances in the study area, depending upon year.

We discriminated between active roads (paved roads and 1^st^ order forestry roads) and potentially derelict roads (2^nd^ and 3^rd^ order forestry roads, [36]) to represent different levels of human disturbance to caribou [37]. Active roads were 35–90 m wide (including right-of-ways), routinely maintained even during winter, and had a life expectancy of at least 10 years (much more for paved roads). Derelict roads were 15–30 m wide (including right-of-ways) with a life expectancy of a few months up to a maximum of 10 years [36]. Derelict roads were not maintained by logging companies after logging operations were completed, but were likely maintained due to frequent use by hunters and recreationalists. We separated active and derelict roads to test for potential differences in the response of caribou to their different characteristics and local densities (0.19 *vs*. 1.47 km•km^−2^ for active and derelict roads, respectively). We obtained densities of active and derelict roads by dividing the cumulative road length within caribou home ranges by their area (km•km^−2^). We standardized raw values of road densities using the equation ([*x* – μ]/σ), where μ was the mean and σ the standard deviation of the entire range of values *x*.

### Scales of Analyses

We investigated the relationship between the likelihood that a caribou died from predation and their habitat selection pattern, specifically examining their avoidance of anthropogenic sources of disturbance at various spatio-temporal scales [18]. For our broad scale analyses, we assessed survival probability and home range composition using the annual home range delimited with the 100% minimum convex polygon encompassing caribou locations collected during a year (January 1 to December 31, *n* = 245). At the intermediate scale, we used the same approach to determine survival probability and seasonal home range composition (*n* = 874) for five biologically meaningful periods delineated by Courtois [33]: spring (15 April–20 May), calving (21 May–20 June), summer (21 June–14 September), rut (15 September–31 October), and winter (1 November–14 April). However, seasonal home range analyses gave similar results to the annual home range analyses, therefore we only report annual home range results.

For our finest scales of analyses, we examined if the habitat selection patterns of individuals that died from predation differed from individuals that survived, only using data directly preceding the death of individuals. These analyses were performed on 3 separate scales encompassing the last 15, 10, or 5 days before death. Preliminary analyses performed using larger (30 days) or smaller (2 days) time windows had results similar to the 15 days or 5 days analysis, respectively. For these fine-scale analyses, we summarized habitat covariates within buffer circles having a radius of 1 km (and an area of 3.1 km^2^) around each location. We used this method to evaluate the landscape context surrounding each caribou before its capture by predators.

### Statistical Analyses – Broad Scale

We investigated the influence of vegetation, cutblocks, and roads on the following indices of individual performance: the probability that an adult caribou died from predation, the calving rate of females, the probability that a calf died by predation during its first year of life, and home range size. We also included age and age^2^ as covariates because we expected that fitness indices would be related to age, with prime adults performing better than the youngest and oldest individuals [38]–[39]. For each of these analyses, we built 10 candidate models based on our predictions (Table 1), which we compared using Akaike’s Information Criterion corrected for small sample sizes (AIC_c_; [40]). We used the same candidate models for all analyses.

**Table 1:** Description of the candidate models used to investigate the relationship between habitat selection or home range composition and the probability that adult caribou died from predation, the calving rate of females, the probability that a calf died by predation during its first year of life, and home range size of adult forest-dwelling caribou in the Charlevoix region, Québec, Canada.

Model	Name	Variables (units)	Number of parameters (k)
1	Age	Age (year)+Age^2^	2
2	Roads	Density of active roads+Density of derelict roads (km•km^−2^)	2
3	Habitat class	Percentage of each habitat class (%)	7
4	Recent disturbances	Percentage of cutblocks or natural disturbances ≤5 years old (%)	1
5	Age+Roads	Model 1+ Model 2	4
6	Age+Habitat class	Model 1+ Model 3	9
7	Age+Recent disturbances	Model 1+ Model 4	3
8	Roads+Recent disturbances	Model 2+ Model 4	3
9	Age+Roads+Recent disturbances	Model 1+ Model 2+ Model 4	5
10	Age+Roads+Habitat class (Global)	Model 1+ Model 2+ Model 3	11

The habitat class model includes old mature conifer, wetland, deciduous, recent and old disturbances, regenerating, and other.

For each trait, we ranked the 10 candidate models by calculating the difference in AIC_c_ between each model and the best model (ΔAIC_c_ = AIC_ci_−AIC_cmin_) to select the most parsimonious model. We performed multicollinearity diagnostics using the REG procedure in SAS 9.2 (SAS Institute Inc., Cary, NC, USA), and discarded the open lichen woodlands category from all our analyses because of its high variance inflation value (VIF). After its removal, VIF of other variables was ≤1.72, which allowed for valid statistical inferences [41].

We evaluated the probability of adult caribou dying from predation at a broad scale by estimating Cox regression models [42], using status×time as the dependent variable and covariates included in Table 1 as the independent variables. In this analysis, status indicated whether an individual was alive or dead at the end of the year, and time was the number of days between January 1^st^ (or the start of the monitoring if it started within the given year) and either the death or the censoring of the individual. Censoring occurred when the animal survived the whole year (time = 365), when we lost contact with the GPS collar, when we released live individuals without a collar, or when individuals died from a cause unrelated to predation. We performed these analyses using both VHF data from 1999–2000 and GPS data from 2004–2011, with the PHREG procedure in SAS which allowed staggered entry of collared animals into the sample [43]. We also performed an *a posteriori* survival analysis using the same covariates as the most parsimonious model, where we evaluated the effect of increasing active and derelict road densities in areas of low and high total road density (*i.e.*, active+derelict road densities combined), respectively. Specifically, we added to the model a binary variable indicating whether total road density within a given home range was below or above the median total road density within the home range of all caribou (median = 1.65 km•km^−2^), as well as interactions between this dummy variable and active and derelict road densities.

We assessed the effects of broad scale habitat covariates on our two indices of reproductive success by performing logistic regression models in a GLIMMIX procedure (SAS), using covariates included in Table 1 as the independent variables. For the annual calving rate, we used a binary variable indicating whether females had a calf or not in a given year as the dependent variable. For the survival probability of calves up to 1 year-old, we only kept females that calved, and used a dependent binary variable indicating if a female had a calf that either survived or died in its first year of life. We performed these analyses using data collected in 2004–2007 on 73 female-years.

Finally, to evaluate the impacts of human disturbance on our broad scale index of space use, we performed a linear regression using home range size (km^2^) as the dependent variable and the covariates included in Table 1 as independent variables. We performed this analysis with GPS data from 2004–2011 using the GLIMMIX procedure in SAS.

### Statistical Analyses – Fine Scale

To evaluate whether resource selection behavior at a fine spatio-temporal scale was linked to adult caribou survival probability, we compared resource selection of individuals that died from predation (or from an unknown cause) during the last 15, 10, or 5 days before their death to the resource selection of individuals that survived through the same period. We used a resampling approach to randomly match individuals that died (*n* = 20) to individuals that survived (*n* = 39) during the same period prior to the caribou deaths (adapted from [22]). Therefore, each individual that died from predation was randomly paired to one individual that survived, and the pairing changed in each iteration.

After each iteration, we used the resulting database to fit mixed effects resource selection functions (RSF; [44]) using the GLIMMIX procedure in SAS. We set individual-year as a random intercept. RSFs contrasted habitat covariates within 1 km of recorded caribou locations with those found around a similar number of random locations drawn within the annual home range of caribou, using GPS data from 2004–2011. We tested the effect of all habitat covariates (see the global model of Table 1) and interactions between caribou status (either dead or alive) and each habitat covariate. After 1000 iterations for each of the three time windows, we calculated the mean and 90% confidence limits of the parameter estimates obtained from these 1000 RSFs. Contrary to our broad scale analyses, in which we used 95% confidence limits, we present 90% confidence limits for the fine scale analyses due to the smaller sample size.

## Results

From 1999–2000 and from 2004–2011 inclusively, 12 adult caribou died from confirmed predation events, 4 died from natural accidental causes (*e.g.*, calving, drowning), 3 died from accidents involving humans (*e.g.*, moose hunter mistake, problem during capture), and 15 died from an undetermined cause, for a total of 34 mortalities. From 2004–2007, collared female caribou gave birth to 51 calves, 21 of which (41%) survived their first year of life. All confirmed predation events on adult caribou were caused by wolves, while 95% of confirmed predation events on calves were by black bear (*Ursus americanus*) [32].

### Broad Scale

**Figure 1:**
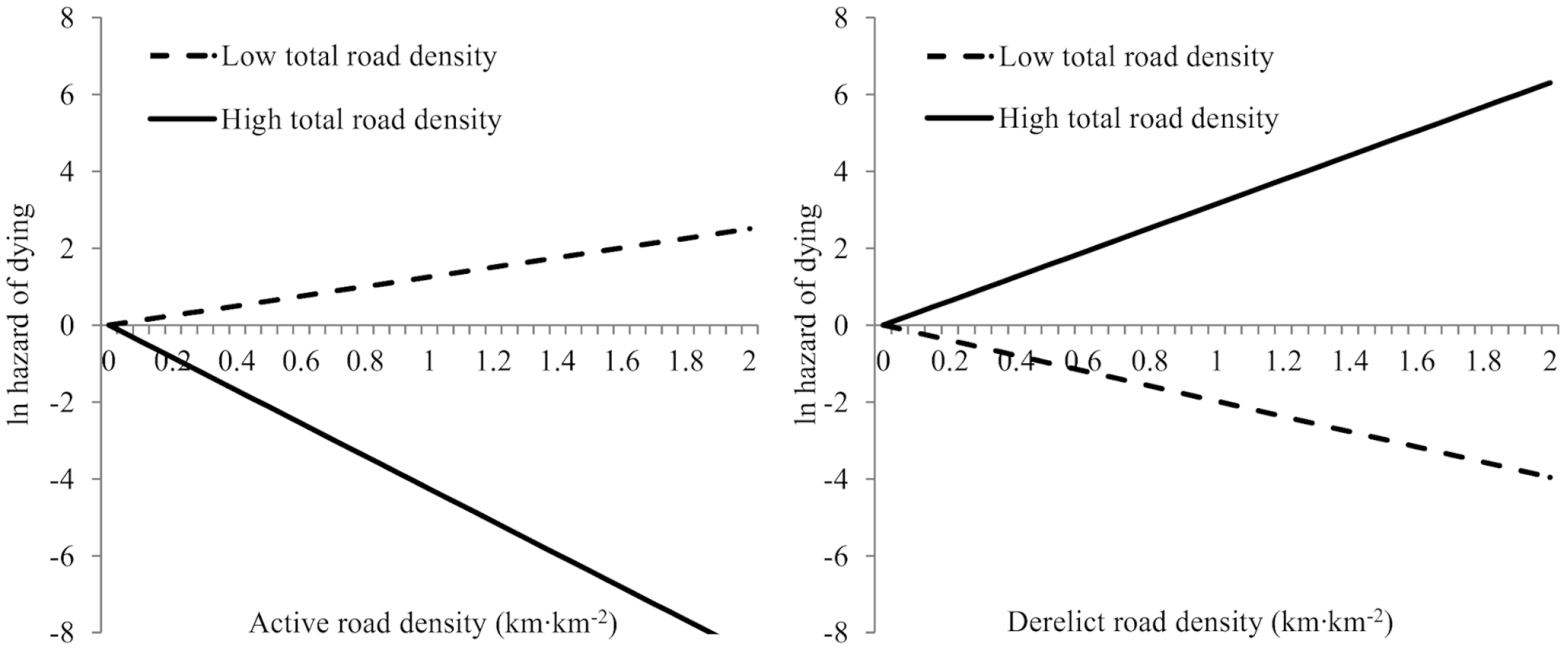
Respective effects of active and derelict road density on the probability that adult caribou are depredated in a given year (represented by the natural logarithm of the hazard of dying) in areas of low and high total road densities (below or above the median total road density found within the annual home range of all caribou), in a population of forest-dwelling caribou in the Charlevoix region, Québec, Canada, from 1999–2000 and from 2004–2011.

Adult survival probability was best explained by a model including the age, road density, and recent disturbances covariates (Table S2). The probability that a caribou died from predation in a given year increased with the proportion of recent disturbances and the density of active roads within their annual home range, and it decreased with the density of derelict roads (Table 2). Our *a posteriori* analysis revealed that the effects of active and derelict road densities on adult caribou survival varied with total road density in their home range (Figure 1). In areas of low total road density, the probability of dying increased with increasing active road density but decreased with increasing derelict road density. In areas of high total road density, both effects were inversed, *i.e.*, the probability of dying decreased with increasing active road density but increased with increasing derelict road density.

**Table 2:** Regression coefficients (β), hazard ratios (HR), and 95% confidence limits of hazard ratios (95% CL) of the most parsimonious models investigating the relationship between annual home range composition and the probability that adults died from predation in a population of forest-dwelling caribou in the Charlevoix region, Québec, Canada, from 1999–2000 and from 2004–2011.

Covariate	β	HR	95% CL
Age (year)	−0.49	0.61	0.36∶1.04
Age^2^	0.03	1.03	0.99∶1.07
*Road density (km•km^−2^)*			
Active roads	0.63	1.88	1.08∶3.24
Derelict roads	−1.03	0.36	0.20∶0.63
*Percentage of habitat class (%)*			
Recent disturbance ≤5 years	0.12	1.13	1.06∶1.21

Results are given for variables included in the most parsimonious models only. HR >1 (with 95% CL excluding 1) indicate an increase in the probability of dying, and HR <1 indicate a decrease of the same probability.

The calving rate of adult females was best explained by a model including road density covariates only, and the probability that a calf died by predation during its first year of life was best explained by a model including recent disturbances only (Table S3). However, the most parsimonious models for both indices of female reproductive success did not include any statistically significant effects (Table 3).

**Table 3:** Regression coefficients (β) and 95% confidence limits (95% CL) of the most parsimonious models investigating the relationship between annual home range composition and the calving rate of females, the probability that a calf died by predation during its first year of life, and home-range size of adults in a population of forest-dwelling caribou in the Charlevoix region, Québec, Canada, from 2004–2011.

Covariate	Calving rate		Calf survival		Home range size	
	β	95% CL	β	95% CL	β	95% CL
Age					0.05	−0.07∶0.16
Age^2^					<−0.01	−0.01:<0.01
*Road density (km•km^−2^)*						
Active roads	0.68	−0.29∶1.65			0.98	0.76∶1.19
Derelict roads	0.03	−0.08∶0.14			0.18	0.16∶0.20
*Percentage of habitat class (%)*						
Old mature conifer					−0.01	−0.03:<0.01
Wetland					−0.01	−0.11∶0.08
Deciduous					0.03	−0.04∶0.10
Recent disturbance ≤5 years			−0.01	−0.11∶0.08	−0.05	−0.07:−0.02
Old disturbance 6–20 years					−0.07	−0.09:−0.05
Regenerating					0.02	−0.01∶0.04
Other					0.08	0.01∶0.15

Results are given for variables included in the most parsimonious models only (otherwise indicated by an empty cell). Statistically significant effects are characterized by 95% confidence limits excluding 0.

The home range size of adult caribou was best explained by a model including age, road density, and habitat class covariates (Table S4). The annual home range area of caribou increased with the density of both types of roads and the proportion of the “other” category, and it decreased with the proportion of recent and old disturbances in the home range (Table 3).

### Fine Scale

The three spatio-temporal scales (15, 10, and 5 days before death) we used to assess fine scale resource selection by adult caribou provided complementary insights on the effects of habitat covariates on adult caribou survival probability (Table 4). Individuals that survived avoided areas with high densities of both types of roads and regenerating stands, and selected recent disturbances and wetlands (during the last 15 to 10 days only; see the estimates for habitat covariates without interaction). The interaction between habitat covariates and caribou status revealed that the selection pattern of individuals that died by predation differed from that of individuals that survived for some habitat variables. Fifteen days before death, caribou that ended up being killed by predators avoided old mature conifer stands, and avoided regenerating stands more than individuals that survived. During the last 10 days, caribou that died selected recent disturbances more than individuals that survived. Finally, individuals that died from predation avoided the “other” category at all spatio-temporal scales, which contrasts with individuals that survived (Table 4).

**Table 4:** Mean (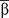) and confidence limits (90% CL) of the parameter estimates of fine scale resource selection functions comparing habitat selection between individuals that died from predation (see estimates of status×habitat covariates interactions) and individuals that survived (see estimates of habitat covariates without interaction) in a population of forest-dwelling caribou in the Charlevoix region, Québec, Canada, from 2004–2011.

Covariate	15 days		10 days		5 days	
	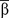	90% CL	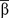	90% CL	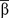	90% CL
Status (Dead)	0.17	−0.28∶0.63	−0.13	−0.58∶0.34	0.04	−0.51∶0.60
*Road density (km•km^−2^)*						
Actives roads	−0.14	−0.23:−0.05	−0.16	−0.27:−0.07	−0.17	−0.30:−0.06
Derelict roads	−0.08	−0.12:−0.05	−0.08	−0.12:−0.05	−0.08	−0.12:−0.04
*Percentage of habitat class (%)*						
Old mature conifer	0.52	−0.04∶1.11	0.46	−0.10∶1.07	0.67	−0.02∶1.36
Wetland	1.37	0.64∶2.00	1.24	0.42∶1.96	1.09	−0.06∶2.00
Deciduous	−3.43	−6.26∶1.54	−3.56	−6.33∶1.24	−4.09	−6.40∶1.56
Recent disturbance ≤5 years	1.83	1.27∶2.38	1.77	1.19∶2.36	1.96	1.25∶2.60
Old disturbance 6–20 years	0.01	−0.88∶0.76	−0.10	−0.93∶0.65	−0.06	−1.12∶0.85
Regenerating	−0.96	−1.80:−0.11	−1.03	−1.94:−0.22	−0.99	−1.96:−0.06
Other	1.13	−0.58∶2.34	0.99	−0.69∶2.24	1.13	−0.80∶2.45
*Interaction between status and road density*						
Status×active roads	0.01	−0.08∶0.10	0.03	−0.06∶0.14	0.07	−0.04∶0.20
Status×derelict roads	−0.01	−0.04∶0.02	−0.01	−0.04∶0.03	−0.01	−0.04∶0.03
*Interaction between status and percentage* *of habitat class*						
Status×old mature conifer	−0.62	−1.20:−0.06	−0.39	−1.00∶0.16	−0.42	−1.09∶0.26
Status×wetland	0.41	−0.22∶1.15	0.56	−0.17∶1.38	0.66	−0.25∶1.81
Status×deciduous	−2.07	−7.06∶0.75	−1.97	−6.82∶0.78	−0.84	−6.35∶1.44
Status×recent disturbance	0.25	−0.30∶0.81	0.64	0.05∶1.20	0.51	−0.12∶1.20
Status×old disturbance	0.04	−0.71∶0.92	0.45	−0.30∶1.28	−0.01	−0.93∶1.06
Status×regenerating	−1.16	−2.02:−0.32	−0.69	−1.48∶0.22	−0.73	−1.65∶0.24
Status×other	−2.12	−3.32:−0.41	−2.60	−3.86:−0.90	−2.46	−3.77:−0.52

We paired individuals that died (*n = *20) with individuals that survived (*n* = 39) during the same time period, and assessed habitat selection within 15, 10 or 5 day periods before the predation event. Estimates of status×habitat covariates interactions represent the difference in selection between individuals that died from predation and individuals that survived, *i.e.*, for individuals that died from predation, the true value of 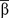 *_x_* (for a given variable *x*) should be calculated as 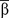 *_x_* of individuals that survived+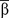 *_x_* of individuals that died. Statistically significant effects are characterized by 90% confidence limits excluding 0.

## Discussion

Interactions between predators, prey, their habitat, and human disturbances in the landscape are complex [45]. The assessment of the fitness consequences of wildlife reactions towards resources and human disturbances require large datasets based on long time series [4]. Our 10 years of spatially explicit data on 87 individual adult caribou allowed us to examine the impacts of commercial logging and roads on the adult survival of a free-ranging large mammal population at multiple scales, while considering other fitness-related traits like reproduction and energy expenditures related to space use. Although many empirical studies found strong negative impacts of human disturbance on the behavior of large mammals (*e.g.*, [2], [9]), assessing the relationship between individual performance and reaction to disturbance was an essential next step to comprehensively assess the real impacts of human disturbance on population dynamics [6].

Our results support the hypothesis that human activities and infrastructure affect individual performance of a large prey species, because of the increased predation risk. Human infrastructure has been shown to influence the behavior of large carnivores [15]–[16], [23] in such a way that anthropogenic disturbances may shape the landscape of fear in which large prey dwell [45]. Roads and cutblocks may have affected survival indirectly by increasing the efficacy of wolves in their search for large prey [16]. Caribou exhibit a strong avoidance towards roads (*e.g.*, [9], [30]), and their vulnerability to predation may be higher in the vicinity of roads [16]. Abundant human infrastructure and disturbed landscapes may modify predator-prey interactions in favor of predators in regions where human development is extensive, and where prey are more sensitive to human disturbance than predators [22]. Our results also suggest that human disturbances may modify the distribution of prey in the landscape by influencing their hierarchical resource selection behavior at both broad and fine spatio-temporal scales [18], [37].

Individuals that established their home range in newly logged areas and/or in areas with high road densities had a much higher probability of dying by predation throughout the year. Moreover, although all caribou selected for recent disturbances at our finer scale of analysis, individuals that were killed by wolves showed both a stronger selection for recently disturbed stands (during the 10 days before death) and an avoidance of mature conifer stands (during the 15 days before death). Recent cutblocks may increase predation risk for caribou through apparent competition, as exemplified by the moose-wolf-caribou interaction in the boreal forest [24], while old mature conifer stands are usually associated with lower predation risk for caribou [46]. Recent work by Dussault et al. [22] and Pinard et al. [32] show that selection for recent cutblocks by female caribou during calving improved reproductive success, but that this behavior became detrimental as regeneration took place. We show here that strong selection for disturbed stands during other periods may be detrimental for the survival of adult caribou, even during the early seral stages ≤5 years following cutovers.

High densities of active roads had the strongest negative impact on adult caribou survival at a broad scale. Our survival analysis showed that an increase of 0.25 km•km^−2^ (*i.e.*, the equivalent of a 1 unit increase in standardized density) of active roads in the annual home range of a caribou would increase its risk of dying by 88%. Such an increase in active road density is substantial, considering that the overall active road density in our study area was 0.19 km•km^−2^. This result demonstrates that the construction of a paved or 1^st^ order forestry road within the home range of a caribou may significantly increase the risk of predation.

However, we also found that the effects of active and derelict road densities on adult caribou survival varied with the overall density of roads in the landscape. The respective effects of active and derelict road densities were reversed at low and high total road densities, with high active road densities being detrimental at low total road density, and high derelict road densities being detrimental at high total road density.

A fundamental aspect of our survival analysis is that it investigates the interplay between two species (*i.e.*, prey and their predators). Therefore, this result may stem from the functional response of wolves to roads, in our study area [47]–[48] as well as in other regions [15]. Lesmerises et al. [47] found that wolves in Charlevoix generally selected high road density areas, whereas Houle et al. [48] found that wolves selected roads more strongly where local road density was low. However, neither study discriminated road types based on their width or respective level of disturbance like we did. Hebblewhite and Merrill [15] found that wolves selected areas close to human infrastructure in areas where human activity was high, but they also showed that the reactions of wolves varied among individuals and packs, and with time of day. Therefore, wolves may have favored one type of road over the other based on their availability in the landscape, which may have modified the respective impacts of active and derelict road densities on adult caribou survival. More research is needed to document the functional responses of wolf to different types of roads, and their subsequent consequences on predation risk for large preys.

Although cutblocks and active roads included in the home range of adult caribou increased their probability of dying from predation, they had no significant effect on indices of reproductive success. Calving rate in the highly fragmented landscape of Charlevoix from 2004–2007 was 80%, which is slightly lower than what was observed in other woodland caribou populations (see, *e.g.*, [13], [33]). Most woodland caribou populations in Canada, even those with negative growth rates, show relatively high gestation rates [49]. Moreover, mortality of caribou calves was not explained by the presence of human disturbances in the annual home range of their mother. Therefore, our results do not support our prediction that female reproductive success would decrease in disturbed regions because of the direct and indirect costs of anthropogenically enhanced predation risk [28].

Conversely, Dussault et al. [22] recently showed that high road densities in areas used by female caribou during the first weeks following calving could influence the survival of their calf through increased bear predation. These authors evaluated the effects of anthropogenic disturbance on reproductive success in the same caribou population, but used a different scale of analysis, which may explain why we obtained conflicting results [18]. The behavioral reactions of female caribou towards roads may affect their reproductive success at a finer spatio-temporal scale than what we used in our study, *i.e.*, within the first 4–6 weeks of the life of their calves [22]. We showed that the same conclusions could not be drawn at the broader scale of the annual home range, and that all scales were not equally important for this particular trait [50]. Despite the lack of evidence that anthropogenic disturbances affected the reproductive success of female caribou at the home range scale, we argue that the effects on adult survival should not be overlooked. Reduced adult survival may have more profound impacts on the population dynamics of caribou and other large herbivores, compared to reduced calf survival [35].

The size of an animal’s home range depends mainly on the interaction between its energetic requirements and the spatial configuration of resources and constraints in its environment [51]. We found that the size of a caribou’s home range increased in areas encompassing high road densities. Roads may have affected home range size by causing functional habitat loss and fragmentation, increasing the distance between suitable habitat patches, or making some resources inaccessible [8]. Conversely, and contrary to our prediction, high proportions of disturbed habitat decreased home range size. Although animals usually increase home range with decreasing habitat quality (*e.g.*, [52]–[53]), large herbivores were also shown to decrease the size of their home range with increasing patch and edge density [54]. Other studies have found that caribou occupied smaller ranges in regions that were strongly impacted by commercial logging activities, possibly to avoid degraded habitat [12], [55]. However, more confined home ranges could have made the detection of caribou more predictable for wolves, and may have compromised the “spacing out” anti-predator strategy of caribou [56], especially in recently disturbed areas (≤5 years old) where we found that the probability of predation increased.

Although we did not investigate the influence of human disturbance on body condition, empirical evidence suggests that prolonged stress caused by human disturbance may affect the growth and body condition of animals [14]. Roads may have indirect impacts on the energetic balance of animals due to increased costs (*e.g.*, higher movement rates, costly behavioral reactions) or reduced food intake [14]. For example, Murphy and Curatolo [57] observed that caribou reduced their food acquisition and showed increased vigilance in the vicinity of roads. In our study, larger home ranges may have increased energy expenditure by caribou, but not sufficiently to affect other activities like reproduction, as demonstrated by the lack of effect of human disturbances on calving rate. More research will be needed to assess whether individual fitness is affected by energy lost through increased movement rates or larger home range sizes in areas of high road densities.

In the last few decades, humans have profoundly modified caribou habitat, and these changes occurred so quickly that behavioral adaptations aimed at avoiding predation risk as well as human disturbances may have been insufficient. This has led to a global decline of caribou populations [20]. Through the creation and application of recovery plans, conservation agencies have been protecting caribou by limiting direct mortality (*e.g.*, by prohibiting caribou hunting) and preserving critical caribou habitat [49]. If caribou are to be maintained in human-modified landscapes over the long term, additional efforts should be directed towards mitigating the direct and indirect impacts of human disturbance on individual performance. Bridging the gap between behavioral reactions and population dynamics should be a goal pursued by researchers working to ensure the conservation of threatened species in this age of global biodiversity decline.

## Supporting Information

Table S1 **Parameter estimates (β) ± standard error (SE) of the logistic regression describing the relationship between the cause of mortality of forest-dwelling caribou monitored using GPS telemetry in the Charlevoix region, Québec, Canada, from 2004–2011, and the age and sex of individuals, time of death (in months), and habitat characteristics included in a 1-km spatial extent around mortality sites.**
(DOCX)

Table S2 **Relative support of models used to investigate the relationship between annual home range composition and the probability that adults died from predation in a population of forest-dwelling caribou in the Charlevoix region, Québec, Canada, from 1999–2000 and from 2004–2011.**
(DOCX)

Table S3 **Relative support of models used to investigate the relationship between annual home range composition and the calving rate of adult females, as well as the probability that a calf died by predation during its first year of life, in a population of forest-dwelling caribou in the Charlevoix region, Québec, Canada, from 2004–2007.**
(DOCX)

Table S4 **Relative support of models used to investigate the relationship between annual home range composition and home range size in a population of forest-dwelling caribou in the Charlevoix region, Québec, Canada, from 2004–2011.**
(DOCX)
