# Supplementary material for: Impacts of Human Disturbance on Large Prey Species: Do Behavioral Reactions Translate to Fitness Consequences?
Source: PLoS One. 2013 Sep 11;8(9):e73695. doi: 10.1371/journal.pone.0073695 (PMC3770704; doi:10.1371/journal.pone.0073695)
Supplement: Table S1 — Parameter estimates (β) ± standard error (SE) of the logistic regression describing the relationship between the cause of mortality of forest-dwelling caribou monitored using GPS telemetry in the Charlevoix region, Québec, Canada, from 2004–2011, and the age and sex of individuals, time of death (in months), and habitat characteristics included in a 1-km spatial extent around mortality sites. (DOCX) [file pone.0073695.s001.docx]

Table S1. Parameter estimates (β) ± standard error (SE) of the logistic regression describing the relationship between the cause of mortality of forest-dwelling caribou monitored using GPS telemetry in the Charlevoix region, Québec, Canada, from 2004-2011, and the age and sex of individuals, time of death (in months), and habitat characteristics included in a 1-km spatial extent around mortality sites

| Variable | β ±SE | P |
| --- | --- | --- |
| Intercept | 0.06 ± 1.25 | 0.96 |
| Age of individual | 0.05 ± 0.05 | 0.39 |
| Sex of individual | 0.16 ± 0.54 | 0.77 |
| Time of death (months) | 0.07 ± 0.17 | 0.69 |
| % of old mature conifer | -0.01 ± 0.02 | 0.53 |
| % of wetland | -0.05 ± 0.05 | 0.38 |
| % of deciduous | -0.99 ± 0.97 | 0.34 |
| % of recent disturbance ≤5 years | 0.01 ± 0.01 | 0.56 |
| % of old disturbance 6–20 years | 0.04 ± 0.04 | 0.34 |
| % of regenerating | 0.06 ± 0.11 | 0.57 |
| % of open lichen woodland | 0.01 ± 0.02 | 0.71 |
| % of other | -0.02 ± 0.03 | 0.49 |
| Road density (km∙km^-2^) | <-0.01 ±<0.01 | 0.58 |

Caribou were either killed by wolf (n=11) or died of undetermined causes likely linked to predation (n=9). Results show that individuals, time of death, and habitat surrounding mortality sites were not statistically different between the two mortality causes (all covariates have P ≥ 0.34)
