# Supplementary material for: Impacts of Human Disturbance on Large Prey Species: Do Behavioral Reactions Translate to Fitness Consequences?
Source: PLoS One. 2013 Sep 11;8(9):e73695. doi: 10.1371/journal.pone.0073695 (PMC3770704; doi:10.1371/journal.pone.0073695)
Supplement: Table S2 — Relative support of models used to investigate the relationship between annual home range composition and the probability that adults died from predation in a population of forest-dwelling caribou in the Charlevoix region, Québec, Canada, from 1999–2000 and from 2004–2011. (DOCX) [file pone.0073695.s002.docx]

Table S2. Relative support of models used to investigate the relationship between annual home range composition and the probability that adults died from predation in a population of forest-dwelling caribou in the Charlevoix region, Québec, Canada, from 1999–2000 and from 2004–2011

| Rank | Model | LL | ∆AIC_c_ |
| --- | --- | --- | --- |
| 1 | Age + Roads + Recent disturbances | -99.60 | **0.00** |
| 2 | Age + Recent disturbances | -105.29 | 7.21 |
| 3 | Age + Roads | -104.70 | 8.12 |
| 4 | Age | -107.07 | 8.73 |
| 5 | Age + Roads + Habitat class (Global) | -98.66 | 11.10 |
| 6 | Age + Habitat class | -104.24 | 17.86 |
| 7 | Roads + Recent disturbances | -133.56 | 63.74 |
| 8 | Recent disturbances | -136.69 | 65.92 |
| 9 | Roads | -138.82 | 72.22 |
| 10 | Habitat class | -136.19 | 77.39 |

Model log-likelihood (LL) and difference in AIC_c_ values relative to the most parsimonious model (∆AIC_c_) are given.
