# Supplementary material for: Impacts of Human Disturbance on Large Prey Species: Do Behavioral Reactions Translate to Fitness Consequences?
Source: PLoS One. 2013 Sep 11;8(9):e73695. doi: 10.1371/journal.pone.0073695 (PMC3770704; doi:10.1371/journal.pone.0073695)
Supplement: Table S3 — Relative support of models used to investigate the relationship between annual home range composition and the calving rate of adult females, as well as the probability that a calf died by predation during its first year of life, in a population of forest-dwelling caribou in the Charlevoix region, Québec, Canada, from 2004–2007. (DOCX) [file pone.0073695.s003.docx]

Table S3. Relative support of models used to investigate the relationship between annual home range composition and the calving rate of adult females, as well as the probability that a calf died by predation during its first year of life, in a population of forest-dwelling caribou in the Charlevoix region, Québec, Canada, from 2004–2007

| Model | Calving rate | | Calf survival | |
| --- | --- | --- | --- | --- |
|  | LL | ∆AIC_c_ | LL | ∆AIC_c_ |
| Age | -44.38 | 7.34 | -34.21 | 1.66 |
| Roads | -40.71 | **0.00** | -34.36 | 1.96 |
| Habitat class | -38.08 | 6.64 | -31.02 | 8.20 |
| Recent disturbances | -44.63 | 5.65 | -34.51 | **0.00** |
| Age + Roads | -40.70 | 4.52 | -34.09 | 6.25 |
| Age + Habitat class | -37.75 | 11.28 | -30.79 | 13.81 |
| Age + Recent disturbances | -44.35 | 9.52 | -34.16 | 3.93 |
| Roads + Recent disturbances | -40.66 | 2.13 | -34.36 | 4.31 |
| Age + Roads + Recent disturbances | -40.64 | 6.78 | -34.07 | 8.79 |
| Age + Roads + Habitat class (Global) | -36.65 | 14.72 | -29.44 | 17.82 |

Model log-likelihood (LL) and differences in AIC_c_ values relative to the most parsimonious model (∆AIC_c_) are given.
