# Supplementary material for: Impacts of Human Disturbance on Large Prey Species: Do Behavioral Reactions Translate to Fitness Consequences?
Source: PLoS One. 2013 Sep 11;8(9):e73695. doi: 10.1371/journal.pone.0073695 (PMC3770704; doi:10.1371/journal.pone.0073695)
Supplement: Table S4 — Relative support of models used to investigate the relationship between annual home range composition and home range size in a population of forest-dwelling caribou in the Charlevoix region, Québec, Canada, from 2004–2011. (DOCX) [file pone.0073695.s004.docx]

Table S4. Relative support of models used to investigate the relationship between annual home range composition and home range size in a population of forest-dwelling caribou in the Charlevoix region, Québec, Canada, from 2004–2011

| Model | Home range size (km^2^) | |
| --- | --- | --- |
|  | LL | ∆AIC_c_ |
| Age | -1305.87 | 163.37 |
| Roads | -1347.04 | 245.69 |
| Habitat class | -1365.96 | 294.18 |
| Recent disturbances | -1415.17 | 379.89 |
| Age + Roads | -1238.55 | 32.93 |
| Age + Habitat class | -1259.11 | 85.00 |
| Age + Recent disturbances | -1302.85 | 159.42 |
| Roads + Recent disturbances | -1345.36 | 244.42 |
| Age + Roads + Recent disturbances | -1236.89 | 31.75 |
| Age + Roads + Habitat class (Global) | -1214.33 | **0.00** |

Model log-likelihood (LL) and differences in AIC_c_ values relative to the most parsimonious model (∆AIC_c_) are given.
